# Desmoplastic Ameloblastoma: A Case Report

**DOI:** 10.5681/joddd.2011.006

**Published:** 2011-03-18

**Authors:** Soheyl Sheikh, Shambulingappa Pallagatti, Isha Singla, Aman Kalucha

**Affiliations:** ^1^ Professor, Department of Oral Medicine and Radiology, M.M. College of Dental Sciences and Research, Mullana, Ambala, Haryana, India; ^2^ Post-graduate Student, Department of Oral Medicine and Radiology, M.M. College of Dental Sciences and Research, Mullana, Ambala, Haryana, India

**Keywords:** Desmoplastic ameloblastoma, odontogenic tumor, stromal desmoplasia

## Abstract

Desmoplastic ameloblastoma is a rare variant of ameloblastoma. Up until now, less than 150 patients have been reported in the literature. We report a case of desmoplastic ameloblastoma in a 45-year-old female with a painless swelling in the left anterior maxillary region. Fine needle aspiration yielded no fluid. Periapical and panoramic radiographs as well as computer tomography scan showed a mixed lesion with multilocular appearance. The present case deserves special importance be-cause of its unfamiliar appearance, potentially aggressive nature and high chances of misdiagnosis. Moreover, the radio-graphic features of this lesion rarely point towards ameloblastoma. A partial maxillectomy for tumor resection was per-formed and the involved teeth were removed. This report is an attempt to help the dental community in developing familiarity with the clinical presentation and at the same time advocating to develop a high index of suspicion in recognizing such cases.

## Introduction

Ameloblastoma is the most common borderline odontogenic tumor of epithelial tissue origin.^1^ It accounts for about 1% of all the cysts and tumors of the jaws and 18% of the various odontogenic neoplasms.^2^According to some authors, it is the second most common odontogenic neoplasm, and only odontoma outnumbers it in frequency of occurrence and there are others who go even further by claiming that excluding odontoma, the incidence of ameloblastoma is at least equal to the incidence of all the other odontogenic neoplasms combined.^3^

Clinically, ameloblastoma is known to occur in three forms: solid or multicystic, unicystic and extraosseous.^4^ Similarly, there are two distinct radiographic patterns of radiolucency, unilocular and multilocular.^4^ Various histological patterns described for this neoplasm include follicular, plexiform, basal, granular and acanthomatous.^5^ Except for histological differences, these patterns have no significant bearing on prognosis.^1,2,5^ However, clinical sub-types like extraosseous and unicystic deserve separate consideration because of their less aggressive behaviour and favourable prognosis.^1^

In recent years, the histomorphological spectrum of ameloblastoma has expanded to include a desmoplastic variant. The first detailed report on the desmoplastic variant of ameloblastoma in the English literature was given in 1984 by Eversole,^6^ who described three cases and called it an 'ameloblastoma with pronounced desmoplasia' The World Health Organization (WHO) classification of odontogenic tumors includes desmoplastic type as a rare variation of ameloblastoma.^7^

Ameloblastoma occurs in all areas of the jaws, but the mandible is the most commonly affected area. Within the mandible, the molar-angle-ramus area is involved three times more commonly than are the premolar and anterior regions combined. Radiographically, the intraosseous ameloblastoma is classically described in dental periapical and panoramic films as a multilocular or 'soap bubble' radiolucency.^3^ Desmoplastic ameloblastoma warrants a separate clinicopathologic entity as it differs strikingly from the other forms of ameloblastoma in its anatomical location, morphology, and radiographic appearance. However, age and sex distribution do not differ from the other types of ameloblastoma. Clinically, this tumor has a predilection for occurrence in the anterior or premolar region of the maxilla or the mandible. It presents radiologically as unilocular or multilocular radiolucencies. In most of the cases, their radiographic appearances are often more typical of the fibro-osseous lesions with well-defined or poorly defined borders. Around the globe, surgeons as well as the radiologists are well versed with the clinicoradiographic features of the common ameloblastomas, however, may ignore this variation.^8^

Histologically, this unusual variant is characterized by extensive stromal collagenization or desmoplasia with small nests and strands of odontogenic epithelium.^9^ The purpose of this article is to report a case of ameloblastoma with the characteristic clinical, radiographic and histological features associated with desmoplastic ameloblastoma and to assist the dental community in better understanding of this variation in odontogenic tumor.

## Case Report

A 45-year-old female presented to the Department of Oral Medicine and Radiology, M.M. College of Dental Sciences and Research, Mullana, Haryana, India with an asymptomatic swelling of spontaneous origin in her left maxilla that had started one year previously. Since then, there had been gradual increase in the size of swelling to its present size. She denied experiencing any bleeding, pain or sensory changes. There was no history of trauma and the past dental/medical history was unremarkable.

The physical examination revealed facial asymmetry due to swelling on the left side of face which was oval in shape and had a smooth surface. The skin over the swelling appeared normal. It was not tender on palpation.

**Figure 1:**
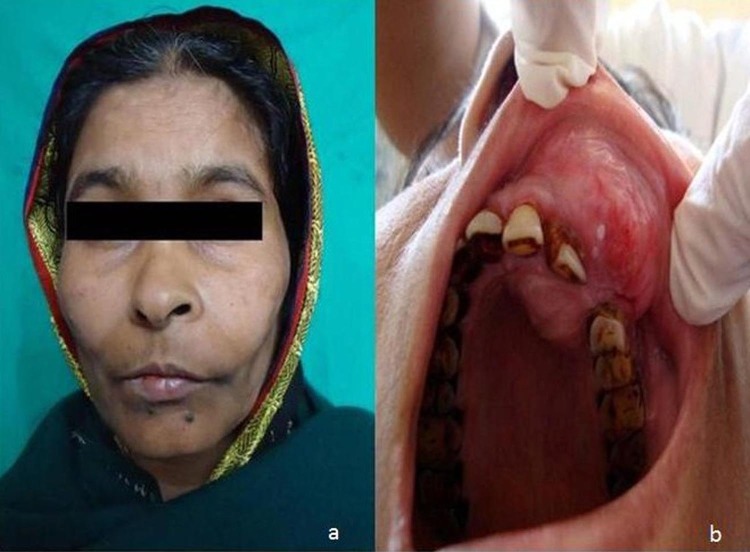
Extra-oral photograph of the patient (a); intra-oral swelling of left maxillary alveolus (b).

The intraoral examination disclosed a large mass, approximately 4.5 × 3 cm in size, extending from the left central incisor to the left first premolar buccally as well as palatally (Figure 1). Buccolingual expansion of the maxillary process was evident. The mucosa over the swelling appeared normal. Also there was a missing maxillary canine on the same side without any history of extraction.

On palpation, the swelling was non-tender and the inspectory findings were confirmed. The swelling was bony hard in consistency, nonfluctuant, nonreducible, noncompressible and nonpulsatile. The teeth in the affected area were not sensitive to percussion and no mobility could be demonstrated. Electric pulp vitality testing revealed that all the teeth in the vicinity were vital except the left upper lateral incisor. No lymphadenopathies or fistulae were present.

**Figure 2:**
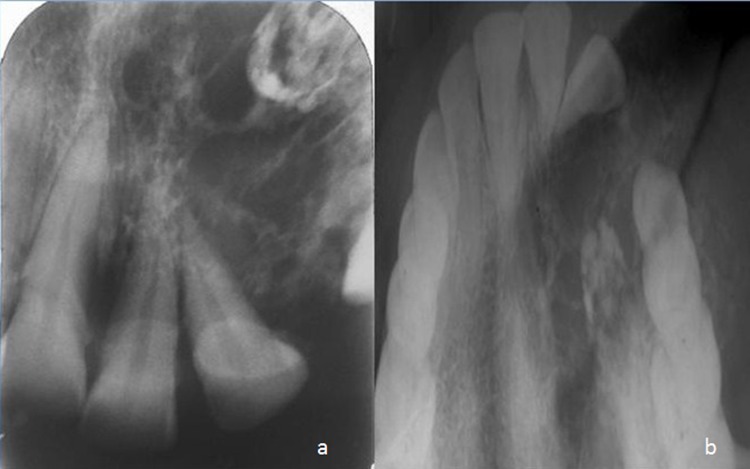
Intraoral periapical radiograph shows an ill-defined multilocular lesion with loss of periodontal ligament space and displacement of lateral incisor (a); maxillary occlusal radiograph showing multilocular appearance and buccal cortical expansion (b).

**Figure 3:**
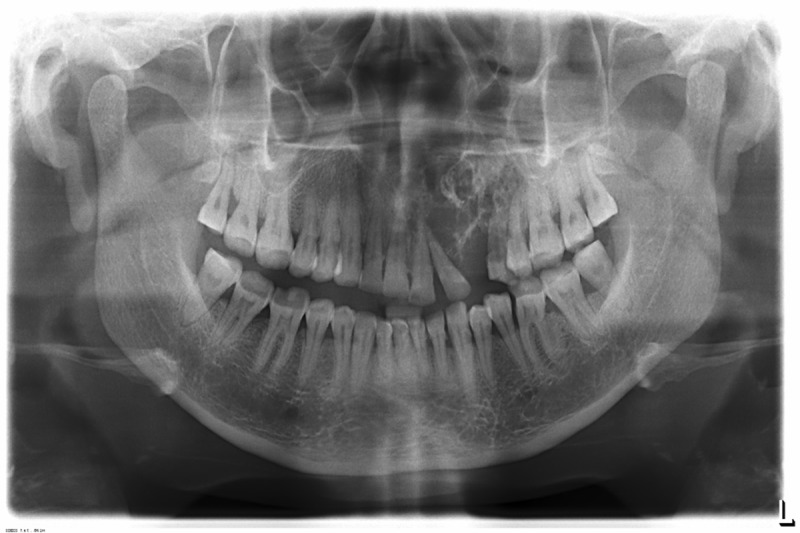
Panoramic radiograph showing mixed ill-defined lesion with flecks of calcification.

**Figure 4:**
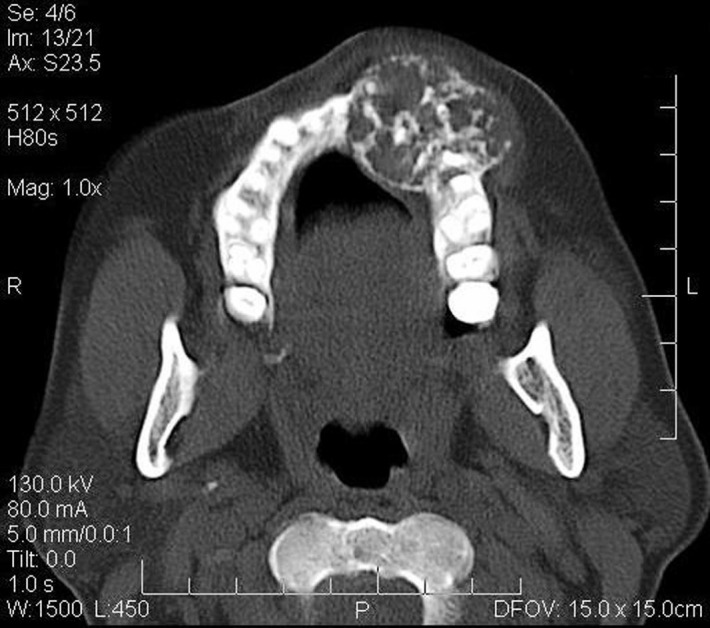
Conventional tomography (CT-scan) axial view showing expansile multiloculated lesion with soap bubble appearance.

Radiographic examination of the maxilla revealed a diffuse ill-defined mixed radiolucent / radiopaque lesion extending from the distal surface of the left central incisor to the mesial surface of the left second premolar, with an approximate size of 2.5 × 3 cm. Small flecks of radiopacities were seen within the lesion. There was loss of lamina dura around the involved teeth. The lesion caused divergence of the root of the lateral incisor, without any signs of root resorption (Figure 2). The floor of the maxillary sinus appeared radiographically intact (Figure 3). Expansion of the buccal cortical plate was evident on maxillary occlusal radiograph. Computed tomography of the lesion showed a predominantly lytic expansile multiloculated lesion, with a size of 3.3 cm mediolateraly, 2.9 cm anteroposteriorly, and 1 cm superoinferiorly, involving left maxillary alveolus. Areas of calcification were present within the lesion giving it a soap bubble appearance (Figure 4).

**Figure 5:**
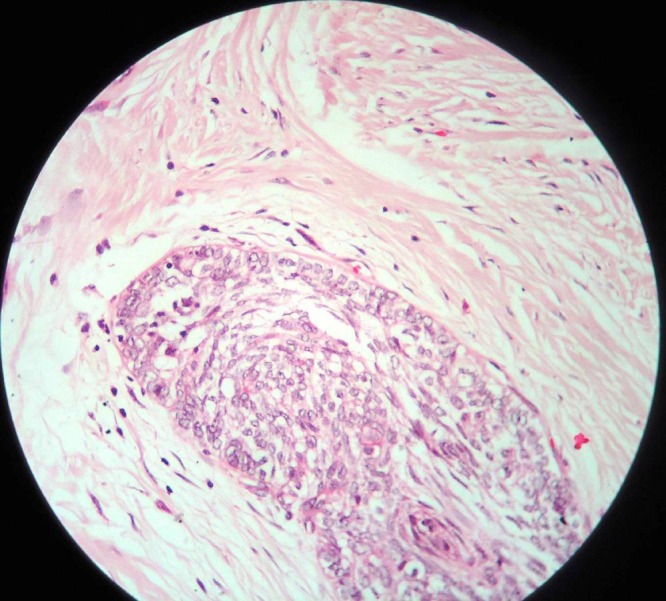
Histopathological image (×40) showing epithelial islands and collageneous stroma.

Based on the clinical and radiographic appearance, a provisional diagnosis of odontogenic myxoma was made. Points in favor of myxoma included its female preponderance, potential to attain considerable size without noticeable signs and symptoms, association with missing tooth (canine in this case), its predilection for premolar–first molar region in the maxilla and diffuse ill -defined borders on radiographs. Aspiration of the lesion was non-productive and a complete hemogram showed values within the normal range. An incisional biopsy was performed under local anesthesia to establish a definitive diagnosis. Histologically, the features were consistent with those of desmoplastic ameloblastoma (Figure 5).

A partial maxillectomy for tumor resection was performed under general anesthesia and a transitional maxillofacial prosthesis was placed. The surgical specimen consisted of a portion of the maxilla and the teeth involved in the tumor. The postoperative course was uneventful and examination of the patient after 1 year showed no evidence of recurrence of the lesion.

## Discussion

In view of the paucity of desmoplastic ameloblastoma case series, it continues to remain one of nature’s secrets. Up until now, less than 150 patients have been reported in the literature. A painless swelling or bony expansion is the most conspicuous clinical manifestation in most of the cases.

Desmoplastic ameloblastoma constitutes 0.9% to 12.1% of all ameloblastomas.The mean age at initial presentation is 42.3 years (range 17-70 years) with age and gender predilection similar to that of other ameloblastomas. Data from different geographical regions seem to suggest a biogeographical pattern in that the relative frequency of desmoplastic ameloblastoma is slightly higher in Asian population. However, more systematic studies on desmoplastic ameloblastoma are needed to prove such suggestions.^8^

Regarding its origin, it is suggested that desmoplastic ameloblastoma develops from the periodontal membrane of the related tooth. Moreover, some suggest that desmoplastic ameloblastoma might arise from epithelial rests of Malassez in the periodontal membrane. In this case, disappearance of the lamina dura and the periodontal ligament space of the adjacent lateral incisor root was clearly identified.^10^

Approximately half of the desmoplastic lesions are located in the maxilla, and the vast majority of them occur in the anterior or premolar portion of the jaws. This is in contrast to the location of the unicystic or classic types of ameloblastoma, which usually are found in the posterior area of the mandible. Maxillary lesions are more insidious than mandibular tumors owing to the proximity of vital structures and the maxillary sinus. Also, the very thin cortical bone of the maxilla forms a weak barrier for the spread of tumors. Consequently, maxillary ameloblastomas may be able to spread earlier and more quickly than do mandibular neoplasms.^11^

Desmoplastic ameloblastoma exhibits a more aggressive behavior than other types of ameloblastoma. This aggressiveness may be due to 1) potential to grow to a large size; 2) the common location in the maxilla leading to an early invasion of adjacent structures; 3) the diffuse radiographic appearance, and 4) histologic finding of bone invasion.^11^ Tooth displacement is a common feature in desmoplastic ameloblastoma in almost 92% of the cases and root resorption is seen in just 33% of the cases.^2^The patient described in this report presented no root resorption, but displacement of the adjacent teeth and also the tumor was associated with a missing tooth.

Radiographically, desmoplastic ameloblastoma usually appears as a mixed radiolucent and radiopaque lesion sometimes mimicking a benign fibro-osseous lesion. The mixed radiographic appearance is due to osseous metaplasia within the dense fibrous septa that characterizes the lesion, and it is not because of the production of a mineralized product by the tumor.^12^ Most of the cases present with poorly demarcated borders. According to Philipsen et al,^13^ radiographically ill-defined borders suggest an infiltrative process with propensity to recur. The panoramic radiograph, conventional computed tomography and magnetic resonance images of desmoplastic ameloblastoma are not specific; they are compatible with those of fibro-osseous lesions and can mimic the radiographic appearance of fibrous dysplasia, chronic sclerosing osteomyelitis, and if well-circumscribed, an ossifying fibroma. Therefore, it is necessary to include these in the differential diagnosis. High resolution bone algorhithm computed tomography images, however, reflect the invasion of tumor elements between peripherally situated bone trabeculae where resorption due to tumor expansion and the deposition of new bone around these resorbed trabeculae has occurred.^14^

In all the reported cases of desmoplastic ameloblastomas, typical radiographic features of ameloblastoma were not observed. The present case, too, lacked typical findings of ameloblastoma and was difficult to diagnose correctly. We suspected fibro-osseous lesion or odontogenic myxoma based on the radiographic findings.

A confirmatory diagnosis of desmoplastic ameloblastoma is made by histopathological evaluation of biopsy specimens. The following features are usually observed during microscopic examination: 1) stromal desmoplasia, in the form of moderately cellular, fibrous connective tissue with abundant collagen, which is the most consistent and distinguishing feature; 2) Islands of different shapes in the epithelial component; 3) peripheral layer of cuboidal cells; and 4) hypercellular central area composed of spindle-shaped or polygonal epithelial cells. Our case was consistent with these common features reported in the literature. Occasionally, desmoplastic ameloblastomas may exhibit interspersed zones of classic follicular or plexiform ameloblastoma; these have been designated as 'hybrid lesions.'^2^

Histologically, the tumor might be misdiagnosed as another odontogenic tumor, as the characteristic palisading layer of ameloblastoma may not be present in all the epithelial clusters, particularly if the biopsy specimen is small. Areas with only narrow strands of epithelial cells within desmoplastic stroma may simulate odontogenic fibroma. The importance lies in the differences in the clinical behavior and management of these two tumors. Ameloblastoma is a potentially aggressive tumor that requires en bloc resection. Odontogenic fibroma is much less aggressive and enucleation is probably curative.^15^

Another differential diagnosis is squamous odontogenic tumor. As this may have a fibrotic stroma, the squamous metaplasia observed in some areas of the desmoplastic variant of ameloblastoma may mimic it if the palisading layer of the tall columnar cells is not identified. Although some cases of squamous odontogenic tumor may have an aggressive clinical course, the currently preferred treatment is curettage, which is followed by few recurrences. Other differential diagnosis includes squamous cell carcinoma and ameloblastic fibroma. The former has prominent cytological atypia while the latter has a cellular stroma. Accurate diagnosis of the desmoplastic variant of ameloblastoma depends on the identification of the typical ameloblastic areas, and this may require examination of more tissue or a repeated biopsy.^15^

Even after two decades of the first report of desmoplastic ameloblastoma, the cause for this peculiar histologic appearance is still unclear. Ng & Siar^16^ using various immunohistochemical techniques demonstrated that the tumor cells of desmoplastic ameloblastoma showed variable expression of S-100 proten and desmin. Similarly, it has been reported that connective tissue stroma in desmoplastic ameloblastoma exhibited a strong positive reaction for collagen type VI.^17^ This was interpreted as an active de novo synthesis of extracellular matrix protein, hence ruling out scar tissue.

With limited understanding of its biologic behavior and prognosis, the proper treatment strategies for desmoplastic ameloblastoma are not entirely defined so far. Resection is the most preferred treatment option for desmoplastic ameloblastoma although some cases were treated by enucleation and/or curettage. However curettage leaves islands of tumor within bone, which later manifest as recurrences. Keszler et al^18^reported a higher recurrence rate (21.4%) for desmoplastic variant than the other types (10.1%) of ameloblastoma.Whether the recurrence is due to the nature of the tumor or due to the incomplete surgery remains speculative. Therefore, block excision is the most widely used treatment to avoid recurrence.

## Conclusion

The desmoplastic ameloblastoma is characterized by specific clinical, imaging, and histological features. For proper understanding of such cases, more in depth analysis and long term follow up is required. The clinician has to be alert regarding the unusual presentation of this neoplasm and should include desmoplastic ameloblastoma as differential diagnosis in any lesion ranging from simple abscess to any fibro-osseous lesions/neoplastic growth presenting in anterior maxilla/mandible. The definite diagnosis requires histopathological examination. Also with the potential for recurrence, such cases should always be treated by complete resection.
